# Beta-Glucan in Foods and Health Benefits

**DOI:** 10.3390/nu14010096

**Published:** 2021-12-27

**Authors:** Seiichiro Aoe

**Affiliations:** The Institute of Human Culture Studies, Otsuma Women’s University, Chiyoda-ku, Tokyo 102-8357, Japan; s-aoe@otsuma.ac.jp

Many articles and manuscripts focusing on the structure, function, mechanism of action, and effects of β-glucan have been published recently. Beta-glucan is a general term for polysaccharides that consist of β-bonds. Structural studies report that combinations of β-1,3 and β-1,6 bonds form long linear β-glucans, and these structures can be detected by specific intestinal receptors, such as dectin-1, which then stimulate the immunological system [1]. Cereal β-glucans that have been derived from barley and oats have also been widely researched in both animal and human studies [2,3,4,5]. They are water soluble, viscous polysaccharides with a linear structure in which glucose is bound through β-1,4 and β-1,3 linkages. Many physiological functions, such as anti-obesity effects, reductions in postprandial glucose increases, and the normalization of serum cholesterol levels have been reported [6,7]. The recent interest in barley and oat β-glucans has been sparked by reports discussing their prebiotic action, which is dependent on molecular weight [8,9]. A marketing report discussing the health benefits of the β-glucans in oats and barley products has also been published [10]. Another β-glucan that has been recently reported on is paramylon, a linear β-1,3-glucan in which glucose is β-1,3 bound and that is abundant in Euglena gracilis. It is an insoluble and unfermentable polysaccharide which is reported to have various physiological functions, including anti-obesity effects and anti-diabetic effects, and has been shown to stimulate immune function [11,12].

This Special Issue entitled “β-glucan in foods and health benefits” reports on the health benefits of indigestible carbohydrates with respect to metabolic diseases and immune functions. The effects of β-glucan have been investigated through the use isolated preparations or natural dietary fibers from whole grain cereals and brans, yeasts, or Euglena. This Special Issue includes original research articles that are based on human intervention studies that address the effects of β-glucan on metabolic diseases and immune function-related markers as well as in vitro and in vivo studies. It also reviews the health benefits of β-glucans in humans.
